# Unusual Metastasis of Pancreatic Adenocarcinoma Into the Bladder: Two Case Reports and Literature Review

**DOI:** 10.1155/criu/2369998

**Published:** 2024-11-19

**Authors:** Mohamadhusni Zarli, Joao G. Porto, Ruben Blachman-Braun, Oleksandr N. Kryvenko, Hemendra N. Shah

**Affiliations:** ^1^Kiran C. Patel College of Osteopathic Medicine, Nova Southeastern University, Fort Lauderdale, Florida, USA; ^2^Desai Sethi Urology Institute, Miller School of Medicine, University of Miami, Coral Gables, Florida, USA

**Keywords:** bladder metastasis, hematuria, hydronephrosis, pancreatic cancer

## Abstract

We described two patients diagnosed with rare bladder metastasis (BM) from pancreatic adenocarcinoma, a prevalent neoplastic disease primarily associated with ductal adenocarcinoma. The overall prognosis for those patients with metastasis is very poor, with a 5-year survival rate of < 3%. The scarcity of cases in the literature makes this series a significant contribution as it presents the first documented instance of BM originating from pancreatobiliary ampullary cancer and a rare case associated with the Krukenberg tumor. Additionally, we extensively reviewed the literature on the infrequent metastasis of pancreatic cancer to the bladder and provided details of those nine previously reported cases. Given its unusual nature, this report highlights the importance of considering BM in patients with a history of pancreatic carcinoma who present with new-onset hematuria or upper tract obstruction, stressing the need for comprehensive evaluation and timely management.

## 1. Introduction

Pancreatic cancer is the seventh leading cause of cancer-related deaths worldwide in both genders, with ductal adenocarcinoma representing 90% of cases [1]. Pancreatobiliary ampullary carcinoma (AC), a rare subset of periampullary cancers, exhibits a 5-year survival rate of 27.5% [2]. The overall survival for this subtype is 52.5 months, which is lower when compared with the intestinal subtype (115 months). Metastasis commonly involves the liver, peritoneum, and pleura [2, 3]. Nevertheless, bladder metastasis (BM) is a rare phenomenon, with only nine cases reported in the literature over the last three decades [1, 3–10]. Here, we present two more cases of BM from pancreaticobiliary carcinoma. We reviewed the literature to enlighten urologists about this rare condition.

## 2. Case Presentation

### 2.1. Case 1

**Figure 1:**
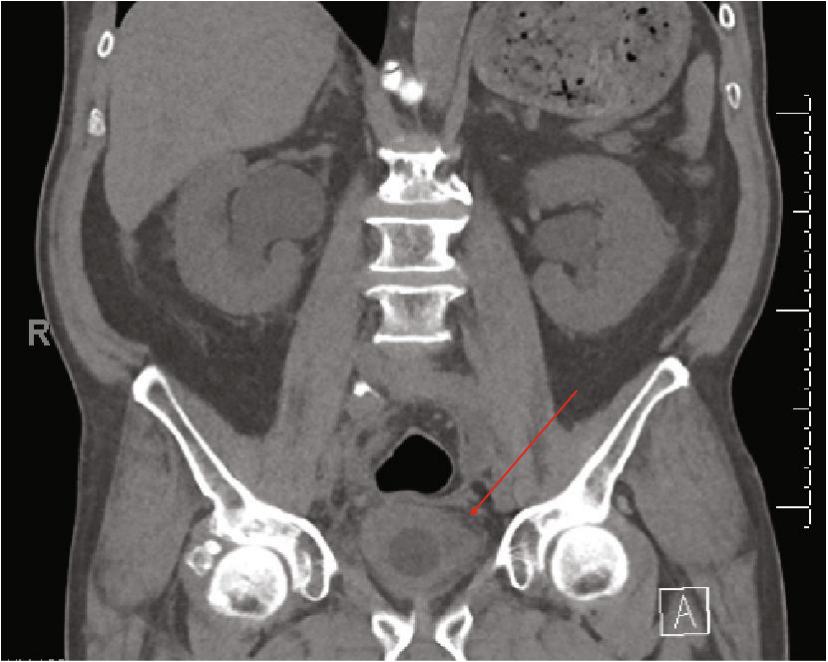
Computerized tomography scan of the pelvis reveals bladder wall thickening (arrow) with perivesicular stranding.

A 74-year-old male with a history of hypertension, hyperlipidemia, diabetes mellitus, a former smoker, and a previous Whipple procedure for a poorly differentiated ampullary adenocarcinoma initially presented to the emergency department (ED) with urinary retention. A Whipple procedure that was done 1 year prior revealed histopathological findings of periampullary/intra-ampullary adenocarcinoma with invasion beyond the sphincter of Oddi, extending into the pancreas and peripancreatic soft tissues and further extending into the duodenal serosa. The patient underwent adjuvant chemotherapy with folinic acid, fluorouracil, and oxaliplatin for 6 months. As this was done at an outside facility, we do not have details of dosages of these drugs. In the ED, he was diagnosed to have a thick wall bladder with bilateral hydroureteronephrosis on a CT scan (Figure 1). There was no obvious tumor recurrence seen intra-abdominally.

**Figure 2:**
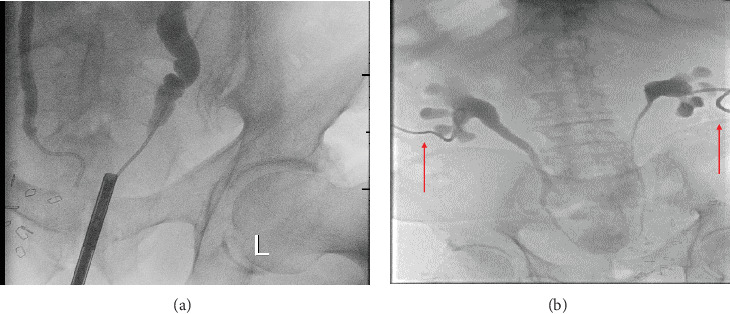
(a) Bilateral retrograde pyelogram (RPG) revealed obstruction at the level of the intramural ureter. (b) A stent malfunction was suspected, and the patient had a bilateral nephrostomy tube (NPT) placed by interventional radiology.

During the hospital stay, his creatinine increased from 0.9 to 2.45 mg/dL, and he underwent a cystoscopy, which revealed a diffuse erythematous bladder wall without any obvious tumor. A bilateral retrograde pyelogram (RPG) revealed obstruction at a level of the intramural ureter, and a bilateral ureteral stent was placed (Figure 2(a)). The creatinine returned to normal after stent placement, but 6 weeks later, the patient developed sepsis and acute kidney injury (AKI) with a creatinine level of 1.38 mg/dL. A stent malfunction was suspected, and the patient had a bilateral percutaneous nephrostomy tube (NPT) placed by interventional radiology (Figure 2(b)). During this admission, he also had abdominal pain, emesis, and constipation and was diagnosed with obstruction at the gastrojejunostomy site due to local tumor recurrence. He was treated endoscopically by placement of an AXIOS stent between the stomach and afferent jejunal limb.

**Figure 3:**
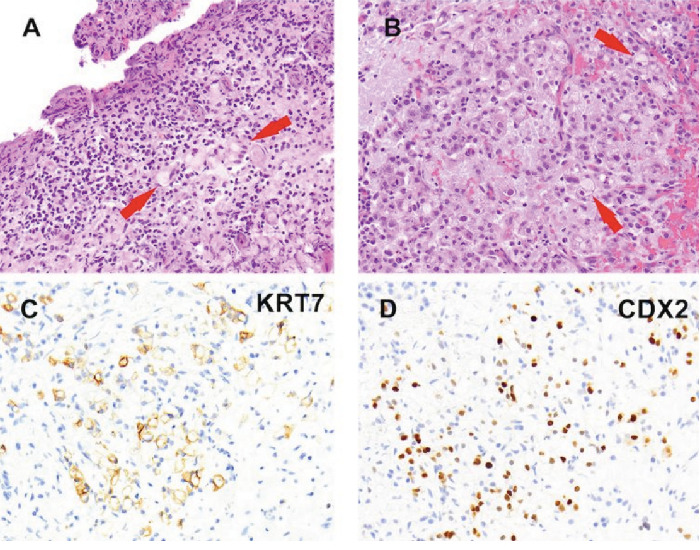
(A) First bladder biopsy showing rare tubular structures lined by relatively bland columnar epithelium containing luminal mucin (arrows). A diagnosis of nephrogenic adenoma was considered. (B) Repeat biopsy shows a more extensive cancer with the presence of signet ring cells (arrows). The cancer is positive for (C) Keratin 7 and (D) CDX2.

One month later, the patient was readmitted with worsening right upper quadrant abdominal pain, nausea, reduced appetite, and lack of bowel movements for 2 days. His CT scan revealed a large bowel obstruction at the distal ascending colon level with signs concerning cancer recurrence. He failed conservative management for bowel obstruction and was subjected 15 months after a Whipple procedure to laparotomy, excision of metastatic mass, and diverting loop ileostomy. Although the patient had multiple peritoneal and retroperitoneal metastases, the obstruction was possibly related to a large peritoneal mass. During the procedure, urologists were consulted with concern for bladder mass. A cystoscopy was done and revealed a small-capacity trabeculated bladder with an obstructive prostate. He underwent a posterior wall bladder biopsy with bipolar cautery, revealing scant epithelial elements forming tubules with intraluminal mucin. Significant crush and cautery artifacts were present, hampering interpretation. A diagnosis of nephrogenic adenoma was considered, but attempts to confirm the diagnosis with immunohistochemistry were unsuccessful because of a limited amount of tissue (Figure 3(A)) [5]. The biopsy of peritoneal nodules revealed metastatic adenocarcinoma.

**Figure 4:**
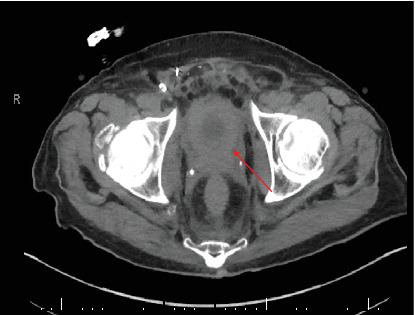
Computerized tomography scan of the pelvis reveals a markedly thickened, small bladder (arrow) with a prostate of 22 g.

He was then scheduled after another month for a stent exchange with the goal of removing NPTs. His repeat CT scan reported that both the mid and distal ureters were not well delineated due to surrounding band-like soft tissue stranding along the retroperitoneum with circumferential thickening of the rectal wall (Figure 4). The prostate measured 22 g on a CT scan. The patient underwent a rectal exam, which revealed a firm consistency of the prostate. The prostate-specific antigen (PSA) level was measured at 1.6 ng/dL. Further examination showed narrowing of the entire right ureter and the lower one-third of the left ureter. To address this issue, bilateral metal ureteral stents were inserted following ureter dilation. Additionally, bladder mucosal and detrusor muscle biopsies were performed using bipolar cautery, while prostatic biopsies were obtained under transrectal ultrasound guidance. The volume of cancer sample by this procedure was more abundant, showed the presence of signet ring cells (a feature not permissible within a nephrogenic adenoma) [11], and allowed us to classify this tumor with appropriate immunohistochemical analysis as metastatic poorly differentiated pancreaticobiliary adenocarcinoma involving bladder and prostate (Figures 3(B), 3(C), and 3(D)). Remarkably, histologically, this tumor was identical to the one responsible for causing a previous large bowel obstruction.

Considering the presence of metastatic disease, the patient was advised to keep the NPTs in place. However, he continued to have multiple episodes of bowel obstruction and sepsis. As a result, the patient was not considered a candidate for palliative chemotherapy, and the decision was made to transition the patient to palliative hospice care.

### 2.2. Case 2

**Figure 5:**
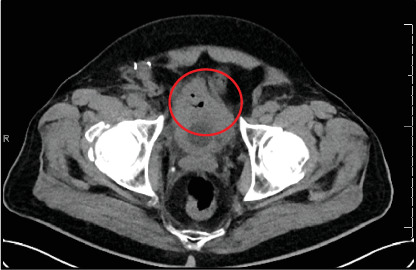
Computerized tomography scan of the pelvis reveals a markedly thickened, small bladder (circle).

A 78-year-old female presented postmenopausal vaginal bleeding. A transvaginal ultrasound was done and revealed a 10.4 × 9.5 × 12.8 cm complex mass overlying the right adnexa. A subsequent CT scan done 3 months later demonstrated disease progression with a right adnexal mass measuring 17 cm, associated with ascites and suspicious omental disease. An exploratory laparotomy was planned with suspicion of primary ovarian cancer, but due to the development of bilateral deep venous thrombosis, the patient required an inferior vena cava filter placement, delaying the scheduled surgery. Exploratory laparotomy with total abdominal hysterectomy and bilateral salpingo-oophorectomy with omentectomy was eventually performed 8 months after her initial presentation. Surprisingly, the biopsy report indicated the presence of pancreatic adenocarcinoma cells in the right ovary, which confirmed a diagnosis of Krukenberg tumor. A subsequent CT scan with contrast done 3 months postoperatively revealed a 6.2 × 2 cm hypoenhancing area in the pancreatic body and tail, which was conspicuously absent on the initial CT scan. Endoscopic biopsy further confirmed the moderately differentiated primary pancreatic adenocarcinoma. The patient was given chemotherapy with gemcitabine 1.4 g and paclitaxel 141 mg. Five months into the treatment, a follow-up CT scan demonstrated disease progression, with the identification of a new mass of 1.1 × 1.1 cm at the dome of the urinary bladder (Figure 5). She continued chemotherapy with gemcitabine 1.4 g and paclitaxel 141 mg. At 18-month follow-up, the patient completed 17 cycles of her chemotherapy and remained asymptomatic. Her CT scan revealed a stable 2.7 × 1.8 cm hypodense mass in the pancreatic body with atrophy of the distal pancreatic body and tail and the absence of a previously seen lesion on the bladder dome.

## 3. Discussion

Infrequently, the urinary bladder can be affected by secondary tumors through direct extension or metastasis from other organs [6]. The most common secondary tumors involving the bladder are prostate (men), uterine cervical (women), and colorectal cancer (both genders). While BM are rare, comprising < 1% of all bladder neoplasms, they represent a significant clinical challenge due to the difficulty in diagnosis and management [6, 7]. BM usually occurs due to malignant tumors originating in the pelvic organs, such as urogenital, colon, and rectal cancer. Among the various malignancies that can spread to the bladder from distant organs, common types include malignant melanoma, breast cancer, and stomach cancer [8].

The incidence rate of BM from primary pancreatic malignancy is extremely low, reported at 0.5% [6]. Mechanisms underlying this rarity remain unclear, potentially attributed to factors such as the muscular nature of the bladder, lymph vascular distribution, and microenvironmental milieu [9]. Involvement of the urinary tract in the settings of pancreatic carcinoma has been described as causing displacement of the kidneys, which is far more frequently observed than direct invasion or metastasis to the renal parenchyma. Ureteral obstructions and encasement by tumor, as seen in our first case, have been reported earlier [2]. However, metastasis to the bladder is exceptionally rare and is usually detected at advanced stages with peritoneal seeding. The dissemination of cancer cells to the bladder can occur through hematogenous spread or “drop metastases.” Bladder wall metastases are typically small and do not cause noticeable symptoms like hematuria unless there is ulceration of the overlying bladder mucosa [6].

The metastatic spread of pancreatic cancer is influenced by several factors, including biological tumor behavior, lymphatic drainage patterns, and the patient's overall condition. The Whipple procedure alters the anatomy of the upper gastrointestinal and pancreaticobiliary regions and theoretically could influence metastatic patterns due to changes in lymphatic and blood flow dynamics following the removal and reconstruction of gastrointestinal tissues. Additionally, surgical manipulation and potential residual microscopic disease could also influence the pathways of metastatic spread. In Case 1, metastases were observed in peritoneal and retroperitoneal locations, which could be attributed to the dissemination of tumor cells along altered lymphatic routes or direct seeding during surgery. However, none of the previously reported cases have established an association between the Whipple procedure and BM. In some instances, the diagnosis was made even before any surgical intervention.

Given the rarity of BM from pancreatic cancer and the limited available literature on this topic, we reviewed all related cases that have been reported in the literature over the last three decades since 1992 (Table 1 [1–3, 5–10]). We found a total of nine patients, including the present report. The previous literature reports that patients ranged in age from 41 to 90 years old, with a female predominance (66.6%). The primary cancer location was predominantly the head (62.5%), followed by the body (25%), and the tail (12.5%). The BMs were located mostly in the posterior wall (77.8%), with associated hematuria as the initial presentation in all but two patients. Peritoneum was the most common site of other metastases (55.6%), followed by the liver (22.2%), lungs (22.2%), spine (11.1%), umbilicus (11.1%), and adrenal gland (11.1%). Most patients developed BM several years after the primary pancreatic cancer diagnosis, with the shortest timeline being a simultaneous presentation.

Compared to the observations reported in the literature, our first patient had BM 1 year after his initial diagnosis of pancreatic cancer, with metastasis also found in the peritoneum, umbilicus, and prostate gland. It also represents a distinct case of BM from ampullary cancer, which is different from the commonly reported pancreatic ductal adenocarcinomas. To the best of our knowledge, this is the first reported instance of BM originating from a pancreaticobiliary AC and the only case that involved diffuse thickening of the entire bladder wall akin to linitis plastica of the bladder as opposed to the reviewed cases in Table 1. It was initially misdiagnosed as a nephrogenic adenoma due to the limited amount of tissue and low volume of the specimen. Although not done in our patient, the use of urine cell blocks might help in improving the diagnostic rate in such cases. In our second case, pancreatic cancer was initially not observed on a CT scan, leading to clinical misdiagnosis of primary ovarian cancer. It was only after the omentectomy that metastatic cancer to the ovaries from pancreatic cancer was diagnosed. There are only 32 cases reported in the literature wherein pancreatic cancer metastasized to the ovaries [12]. Similar to most cases described previously, most of these patients are misdiagnosed as having primary ovarian cancer. The tumor progressed despite chemotherapy with the development of BM [12]. This is likely to be a “drop metastasis,” as reported earlier by Shah et al. [3].

## 4. Conclusion

Pancreatic cancer is a highly aggressive malignancy, and while the most common sites of metastasis are the liver, lung, and bone, it is crucial not to overlook uncommon sites such as the bladder. Metastasis may indicate a poor prognosis for this disease. BM can be asymptomatic in the early stages and progress rapidly thereafter. Symptoms may include hematuria, urinary retention, dysuria, and hydronephrosis. Diffuse thickening of the bladder on CT imaging should prompt physicians to investigate BM. Timely detection and diagnosis may aid in improved patient care.

## Figures and Tables

**Table 1:** Literature review of all cases since 1992 of pancreatic cancer metastasis to the bladder with two reported additional cases.

**Author**	**Year**	**Age, gender**	**Primary site of cancer**	**Features and site of BM**	**Urinary symptoms**	**Other sites of metastasis**	**Time for BM diagnosis after PC identified**
Chiang, Lamki, and Athey [8]	1992	64, female	Head	Thickening of right posterior wall (CT)	Hematuria	Peritoneum and liver	4 years after the excision of the primary tumor
Lavelle, Williams, and O'Leary [5]	2011	84, female	Head	Single mass in posterior wall (CT)	Hematuria	Liver, lungs, spine, and umbilicus	6 years after
Petrides, Singh, and Hodgson [6]	2013	80, female	Head	Uniformly inflamed bladder (US) Papillary mass in posterior wall (cystoscopy)	Hematuria	Lungs	4 years after
Cellini and Deighton [9]	2014	52, female	Tail	Mass in posterior wall (CT) Pedunculated mass in posterior wall (cystoscopy)	Hematuria and dysuria	Peritoneum	21 months after
Shah et al. [3]	2018	66, female	Head	Single mass in the dome (CT)	None	Peritoneum	Simultaneous
Lacombe et al. [10]	2018	76, female	Body	Single lesion (11 mm) in the left posterior wall (CT)	None	Lungs and peritoneum	2 years after
Pike et al. [2]	2020	41, female	Tail	Thickening of posterior wall (CT)	Hematuria and bilateral hydronephrosis	Peritoneum	NA
Arcovito et al. [1]	2020	76, male	Head	Thickening (5 mm) in the base (US)	Hematuria	Invasion of superior mesenteric vein	8 months after
Kubota et al. [7]	2021	90, female	Body	Single mass (15 mm) in posterior wall (cystoscopy)	Hematuria	Adrenal gland	Simultaneous
Case 1	2022	74, male	Ampulla/periampullary	Diffuse thickening of bladder wall (CT)	Urinary retention, bilateral hydronephrosis, and hematuria	Peritoneum, umbilicus, and prostate	1 year after
Case 2	2022	78, female	Body and tail	Single mass in the dome (CT)	None	Ovarian	6 months

Abbreviations: BM, bladder metastasis; CT, computerized tomography; NA, not assessed; PC, pancreatic cancer; US, ultrasound.

## Data Availability

The clinical data used to support the findings of this case report are included within the article.
